# On the Comparison of Incompatibility of Split Systems Across Different Numbers of Taxa

**DOI:** 10.1007/s11538-021-00911-6

**Published:** 2021-05-21

**Authors:** Michael Hendriksen, Nils Kapust

**Affiliations:** Institut für Molekulare Evolution, Heinrich-Heine Universität, Düsseldorf, Germany

**Keywords:** Phylogenetic trees, Compatibility, Split systems, Bipartitions, Matchings, SplitsTree, Archaeal genomes

## Abstract

We consider the problem of the minimum number of phylogenetic trees it would take to display all splits in a given set, a problem related to *k*-compatibility. A set of trees that displays every single possible split is termed a *universal tree set*. In this note, we find the universal incompatibility *U*(*n*), the minimal size of a universal tree set for *n* taxa. By normalising incompatibility using *U*(*n*), one can then compare incompatibility of split systems across different numbers of taxa. We demonstrate this application by comparing two SplitsTree networks derived from archaeal genomes, with different numbers of taxa.

## Introduction

Phylogenetic trees are ubiquitously used to represent the evolutionary history of organisms (Felsenstein 2004). Each edge in an unrooted phylogenetic tree corresponds to a bipartition of the taxa set, and a given phylogenetic tree can be uniquely identified with the set of bipartitions induced by its edge set (Buneman 1971). However, data can often produce conflicting results, whether through measurement error or complex biological phenomena such as incomplete lineage sorting or lateral gene transfer. This can result in splits that contradict each other.

This naturally gave rise to the concept of *k*-compatibility [(first studied by this name by Dress et al. (2001), originally studied as *k*-cross-free families by Karzanov and Lomonosov (1978)], which, given a set of splits *S*, asks for the maximum size of a subset of *S* in which any two splits are incompatible with each other. If this subset is of size *k*, the split system is termed *k*-compatible. We consider a related concept of incompatibility which is arguably more natural —that of the smallest number of phylogenetic trees it would take to display all splits indicated by the data, a so-called *minimal tree set*. In the case that *S* is the set of all possible splits for a set of taxa *X*, we say that a set of phylogenetic trees that display all splits in *S* is a *universal tree set*.

In the present paper, we consider the question of maximum possible incompatibility in this way, that is, given a set of taxa *X* of size $n \ge 2$, how large is a universal tree set of minimum size? We term this *universal incompatibility* and denote it by *U*(*n*). This can also be characterised as finding the minimum $k \ge 1$ such that every split system *S* on *X* can be displayed by *k* phylogenetic trees.

By characterising *U*(*n*), one can then contextualise a split system in terms of how incompatible it is compared to the worst case scenario—that is, the scenario in which every possible split is contained in our split system. Further, by normalising the minimal tree set size using *U*(*n*), we can then compare incompatibility of split systems across different numbers of taxa.

Of particular interest is the fact that the widely-used SplitsTree software (Huson and Bryant 2006) creates a so-called *split network*, which is used to represent conflicting split signals from data. The present results will now allow those who use SplitsTree to fairly compare incompatibility of data across different numbers of taxa.

In Sect. 2, we provide background information. In Sect. 3, we prove some lemmas on bipartitions. In Sect. 4, we apply these lemmas and some classical theorems to prove the main result. In Sect. 5, we then apply these results to compare the incompatibility of two SplitsTree networks of different sizes derived from archaeal genomes.

## Background

A phylogenetic tree on a set of taxa *X* is a connected acyclic graph (*V*, *E*) such that there are no vertices of degree 2 and the degree-1 vertices (termed *leaves*) are bijectively labelled by the elements of *X*.

Recall that a *split* *A*|*B* of a set *X* is a bipartition of *X* into two non-empty sets *A*, *B*; where $B = X \backslash A$. Define the *size* of a split *A*|*B* to be $\min (|A|,|B|)$. We denote by ${\mathcal {S}}(X)$ the set of all splits of *X*, and any subset *S* of ${\mathcal {S}}(X)$ is called a *split system* on *X*. Any split of size 1 is termed *trivial*.

Given a phylogenetic tree $T=(V,E)$ on *X*, each edge can be associated with a split in the following way. If an edge *e* is deleted from *T*, this disconnects the graph into two components, each with at least one labelled vertex. This naturally induces a bipartition on the leaf set *A*|*B*, which we call the split *associated with* *e*, and we say that *A*|*B* is *displayed* by *T* if it is associated with some edge of *T*. In this way, we can consider the split system *induced by* a tree *T*, that is, the set of all splits displayed by *T*. Furthermore, if ${\mathcal {T}}$ is a set of phylogenetic trees on *X*, we say that a split *A*|*B* is *displayed* by ${\mathcal {T}}$ if *A*|*B* is displayed by at least one tree in ${\mathcal {T}}$.

It is well-known (Buneman 1971) that two splits *A*|*B* and *C*|*D* on the same set *X* can only be displayed by the same phylogenetic tree if either one or two of the four intersections

$$\begin{aligned} A \cap C, A \cap D, B \cap C, B \cap D \end{aligned}$$

is empty (noting that if two intersections are empty, *A*|*B* and *C*|*D* represent the same split). If this condition is met by each pair of splits in a split system *S*, we say that *S* is *pairwise compatible*, and if not, the split system is termed *incompatible*. Note that a split is always considered to be pairwise compatible with itself. In fact, *S* is pairwise compatible if and only if *S* corresponds to a phylogenetic tree in the following way.

### Theorem 2.1

(Splits Equivalence Theorem, Buneman (1971)) Let *S* be a collection of splits on *X*. Then, *S* is the split system induced by some phylogenetic tree *T* on *X* if and only if *S* contains all trivial splits on *X* and *S* is pairwise compatible. The tree *T* is unique up to isomorphism.

We will therefore henceforth consider a phylogenetic tree and the corresponding pairwise compatible split set as interchangeable.

In a biological context, sets of incompatible splits frequently arise from data, and biologists wish to quantify the extent to which the set is incompatible. This naturally gave rise to the definition of *k*-compatibility. We say that a split system is *k*-*compatible* if it does not contain a subset of $k+1$ pairwise incompatible splits (for $k \ge 1$). A related concept is that of a *minimal tree set* for a given split system, which we will introduce after defining tree sets. Given a split system *S*, we say it has a *tree set of size* *k* if there exists a set of $k \ge 1$ phylogenetic trees ${\mathcal {T}}$ on the same set *X* such that every split in *S* is displayed by at least one phylogenetic tree in ${\mathcal {T}}$. We say that a set of *k* phylogenetic trees ${\mathcal {T}}$ that displays every split in a split system *S* is *minimal* with respect to *S* if there are no sets of $k-1$ phylogenetic trees with this property.

**Fig. 1:**
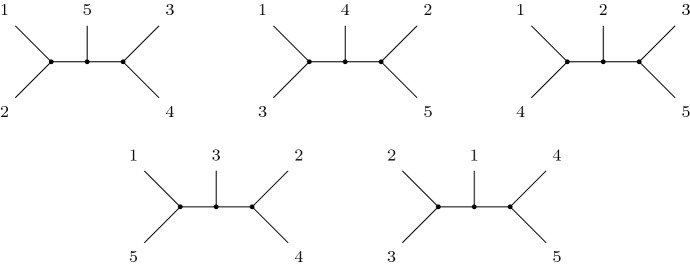
A minimal universal tree set on the 5-leaf set $\{ 1,2,3,4,5 \}$

In the case that $S={\mathcal {S}}(X)$, we say that ${\mathcal {T}}$ is a *universal tree set*. Define the function *U*(*n*) to be the value of $|{\mathcal {T}}|$, where ${\mathcal {T}}$ is a minimal universal tree set on a set of taxa of size $n \ge 2$. An example of a minimal universal tree set for 5 leaves is shown in Fig. 1, showing that $U(5) \le 5$. Indeed, due to the fact that each tree on 5 leaves can display at most two non-trivial splits, and there are ten unique non-trivial splits on 5 leaves, we conclude $U(5) \ge 5$ and thus $U(5)=5$.

We note here that *k*-compatibility of a split system and minimal tree set size of a split system is related concepts. Certainly if a split system is *k*-compatible, the minimal tree set size is at least *k*, as given a set of *k* pairwise incompatible splits each must be displayed by a different phylogenetic tree. Therefore, minimal tree set size is bounded below by *k*-compatibility, but they are not the same, as the following example shows. We thank an anonymous reviewer for this example.

### Example 2.1

Let $X= \{1,2,3,4,5\}$ and let $S = \{12|345,23|145,34|125,45|123, 15|234\}$. Then, for instance, 12|345 and 23|145 are incompatible, but *S* does not contain a pairwise incompatible subset of size 3, so *S* is 2-compatible. However, as a phylogenetic tree with 5 leaves can display at most 2 splits of size 2, the minimal tree set size of *S* is 3.

Finally, we note as an aside that a universal tree set (minimal or otherwise) has no requirement that all phylogenetic trees in the set must be binary. However, given a minimal universal tree set containing a phylogenetic tree *T* that is not binary, one can replace *T* with a binary refinement of *T* (i.e. a binary tree that displays all of the splits displayed by *T*) without compromising minimality of the set—the tree set will still display all splits on *X*. Hence, for a given set *X* there will always exist a minimal universal tree set on *X* consisting only of binary phylogenetic trees. Such a minimal universal tree set will generally not be unique, as any phylogenetic tree that is not binary has several possible binary refinements, but this will not affect the calculation of *U*(*n*).

## Combinatorial Results on Splits

In order to discover how many trees are required to display all of the splits in a set, we will first present some results on the maximum number of splits of the largest possible size that can be displayed by a given tree. We will address this question for even and odd *n* separately, noting that for even ($n=2m$) and odd ($n=2m+1$) the largest possible size of a split is *m*.

### Lemma 3.1

Let $n=2m$ be an even integer, where $m \ge 1$. Then, a phylogenetic tree on *n* leaves displays at most one split of size *m*.

### Proof

Let *A*|*B* and *C*|*D* be a pair of splits displayed by the same phylogenetic tree, of size *m*. Then, by the Splits Equivalence Theorem (Theorem 2.1), *A*|*B* and *C*|*D* must be pairwise compatible, and so one of $A \cap C, A \cap D, B \cap C$ or $B \cap D$ is empty. Without loss of generality, suppose that $A \cap C$ is empty. Then, $A \subseteq (X \backslash C) = D$, but since both partitions are of size *m*, it follows that $A=D$, so $B=C$ and thus, *A*|*B* and *C*|*D* are equivalent partitions. The lemma follows. $\square$

Note that of course a phylogenetic tree need not have any such split, as we can consider the star tree—that is, the tree with only trivial splits—for any number of leaves $n \ge 4$. This Lemma gives a lower bound for *U*(*n*) for even *n*, as phylogenetic trees with an even number of leaves can have at most one split of size $m=\frac{n}{2}$, and $$\frac{1}{2} \left( {\begin{array}{c}n\\ m\end{array}}\right) $$ is the number of such splits for a given *n*. In fact, *U*(*n*) actually equals this lower bound in the even case, as we will see in Theorem 4.1.

### Lemma 3.2

Let $n=2m+1$ where $m \ge 2$ is a positive integer. Then, a phylogenetic tree on *X* with *n* leaves displays at most two splits of size *m*.

### Proof

Seeking a contradiction, let *A*|*B*, *C*|*D* and *E*|*F* be three distinct splits displayed by the same phylogenetic tree, so that $|A|=|C|=|E|=m,|B|=|D|=|F|=m+1$. Then, by the Splits Equivalence Theorem (Theorem 2.1), *A*|*B*, *C*|*D* and *E*|*F* must be pairwise compatible, and in particular, one of $A \cap C, A \cap D, B \cap C$ or $B \cap D$ is empty. As *A*|*B* and *C*|*D* are distinct splits, it must be the case that $A \cap C$ is empty. Therefore, $A \subset D$ and $C \subset B$; in fact, $D = A \cup \{x\}$ for some taxon $x \in X$. This implies that $C=B \backslash \{x\}$.

By similar logic, $F = A \cup \{y\}$ and $E=B \backslash \{y\}$ for some taxon *y* so that $y \ne x$ (since *E*|*F* and *C*|*D* are distinct). But then $C \cap E, C \cap F, D \cap E$ and $D \cap F$ must all be non-empty, which is a contradiction. The lemma follows. $\square$

Lemma 3.2 does not consider the case $n=3$, but in this case one can observe that there are 3 such splits (and that a minimal universal tree set on 3 leaves consists of just the star tree on 3 leaves). Outside of this case, as each phylogenetic tree with an odd number of leaves can display up to two of these splits, a natural follow-up question is whether there are any obstructions to pairing all such splits in this way. That is, if $n=2m+1$, can we partition the splits of largest size into compatible pairs so that each tree in our set displays a unique pair of splits of size *m*?

Fortunately we (almost) can, using the concept of matchings. We will need two definitions before we can see this.

### Definition 3.1

A *matching* *M* of a graph *G* is a set of edges of *G* such that no two edges have a vertex in common. A *defect*-*d* matching *M* is a matching so that all except *d* vertices of *G* have an incident edge from *M*. Defect-0 matchings are also referred to as *perfect* matchings.

Let $m<n$ and *Bip*(*n*, *m*) be the set of splits of the set $\{1,\ldots ,n\}$ of size *m*, noting that that $Bip(n,m)\ne \emptyset$ if $m \ge 1$. We construct the *compatibility graph* *G* of *Bip*(*n*, *m*) in which the vertices are the elements of *Bip*(*n*, *m*) and there is an edge between two vertices if and only if they are compatible, distinct splits. We will then find matchings on the compatibility graph *G*, with the aim of having as small a defect as possible—thus pairing our large splits as efficiently as possible.

Readers familiar with Semple and Steel (2003) will note the similarity between our present definition of compatibility graph and the compatibility graph defined in Sect. 4.5 of Semple and Steel (2003), which is defined in terms of characters. Indeed, our present definition corresponds to the specific case in which the characters are binary and the set of characters corresponds to the split system consisting of all splits of size *m* on a set of size *n*. However, the results discussed in Sect. 4.5 of Semple and Steel (2003) concern finding maximum compatible subsets of a set of characters, which is analogous to the question of finding a subset of a split system which corresponds to a minimal tree set of size 1 with a maximal number of splits. This is an interesting topic area, but orthogonal to the present work.

This will require a graph theoretic result courtesy of Little, Grant and Holton (Little et al. 1975), which itself requires an additional definition.

### Definition 3.2

A graph *G* is said to be *vertex-transitive* if, given any two vertices $v_1$ and $v_2$ of *G*, there is some automorphism $f:V(G)\rightarrow V(G)$ such that $f(v_{1})=v_{2}$ and $f(v_{2})=v_{1}$.

We can now state the following theorem.

### Theorem 3.1

Little et al. (1975) Every connected vertex-transitive graph with an even number of vertices has a perfect matching, and every connected vertex-transitive graph with an odd number of vertices has a defect-1 matching.

Hence, it suffices to show that the compatibility graph *G* of *Bip*(*n*, *m*) is connected and vertex-transitive when $n=2m+1$, recalling that we only need to pair our splits in the odd case. However, Theorem 3.1 differentiates between graphs with even and odd numbers of vertices, and the number of vertices of *G* is $$\left( {\begin{array}{c}n\\ m\end{array}}\right) $$, which can be even or odd depending on the values of *m* and *n*. Therefore, we will need to distinguish these cases. This will require a short detour on the properties of the binomial coefficient, using a theorem of Kummer.

Let *a*, *b* be positive integers, and assume that $a+b$ has *r* digits in base *p*. Then, we can assume $a + b, a,$ and *b* all have *r* digits in base *p* by adding leading 0’s if necessary. Denote the *i*-th digit from the right of *a* and *b* by $a_i$ and $b_i$, respectively. We define a map $\phi : \{1,\dots ,r\} \rightarrow \{0,1\}$ in the following way. Let $\phi (1)=0$ if $a_1 + b_1 < p$, and $\phi (1)=1$ otherwise. Then, for $2 \le i \le r$, we define $\phi (i)=0$ if $a_i+b_i + \phi (i-1) < p$ and $\phi (i)=1$ otherwise. Then, the *number of carries when adding* *a* *and* *b* *in base* *p* is the sum

$$\begin{aligned} \sum _{i=1}^{r} \phi (i). \end{aligned}$$

### Example 3.1

Let $a=15, b=4$, so in base 2 we have that *a* is 01111, *b* is 00100 and $a+b$ is 10011. Then, $\phi (1) = 0$ as $1+0 < 2$, $\phi (2)=0$ as $1+0+0 <2$, and similarly, $\phi (3)=1$, $\phi (4)=1$ and $\phi (5)=0$. Hence, the number of carries when adding 15 and 4 in base 2 is $0+0+1+1+0=2$.

We can now state Kummer’s Theorem.

### Theorem 3.2

(Kummer 1852) If *p* is a prime, then the largest power of *p* that divides $$\left( {\begin{array}{c}m+n\\ n\end{array}}\right) $$, for *m* and *n* non-negative integers, is the number of carries when *m* and $n-m$ are added in base *p*.

As a simple corollary to this, by considering base 2 we get

### Corollary 3.1

If $n = 2m+1$ for positive integer *m*, then $$\left( {\begin{array}{c}n\\ m\end{array}}\right) $$ is odd if and only if $n=2^i-1$ for some integer $i \ge 1$.

For example, $$\left( {\begin{array}{c}7\\ 3\end{array}}\right) = 35$$ and $$\left( {\begin{array}{c}15\\ 7\end{array}}\right) = 6435$$ correspond to the cases $m=3$ and $m=7$, respectively, and yield odd results as *n* is one less than a power of 2.

We can now state our lemma on pairing splits, which uses the concept of matchings in the proof.

### Lemma 3.3

Let $n = 2m+1$, where $m \ge 2$. Then, the compatibility graph of *Bip*(*n*, *m*) has a defect-1 matching if $n=2^k-1$ for some integer $k >1$, otherwise it has a perfect matching.

### Proof

Let *G* be the compatibility graph of *Bip*(*n*, *m*). By Theorem 3.1, the lemma follows if we can show that *G* is connected and vertex-transitive. We first show that *G* is connected.

Let *A*|*B* and *C*|*D* be a pair of splits of size *m* and let $Int(A|B,C|D) =k$ be the size of the intersection between the part of *A*|*B* with $m+1$ elements and the part of *C*|*D* with $m+1$ elements - without loss of generality supposing they are *A* and *C*, respectively. Additionally, note $k>1$. We claim that either *A*|*B* and *C*|*D* coincide (which occurs if and only if $k=m+1$) or there exists a split *E*|*F* in the same connected component of *G* as *A*|*B* so that $Int(E|F,C|D)>k$. As $m+1$ is finite, this implies that *A*|*B* and *C*|*D* must be in the same connected component, and since *A*|*B* and *C*|*D* were arbitrary, that *G* is connected. It therefore remains to be shown that there exists a split *E*|*F* in the same connected component of *G* as *A*|*B* so that $Int(E|F,C|D)>k$.

Suppose *A*|*B* and *C*|*D* do not coincide and consider the split $A'|B'$ obtained by taking some element $x \in (A \backslash C)$ (which must exist as both *A* and *C* have size $m+1$ and do not coincide), and letting $A'=A \backslash \{x\}$ and $B'=B \cup \{x\}$. Note that in this case, $B'$ is now the part of size $m+1$, and further that *A*|*B* and $A'|B'$ are compatible as $B \cap A'$ is empty (as $A'$ is a subset of $A=X \backslash B$). Hence, *A*|*B* and $A'|B'$ are in the same connected component of *G* — indeed, there is an edge between the).

If $Int(A'|B',C|D)>k$, then the claim is proven by taking $E|F=A'|B'$. Otherwise, $Int(A'|B',C|D)= k' \le k < m+1$, and so $A'|B'$ and *C*|*D* do not coincide. Of course, $k' > 0$ as both $B'$ and *C* again have size $m+1$ and do not coincide. We can therefore take some element $y \in B \cap C$ and form the new split $A''|B''$ (also of size *m*), where $A'' = A' \cup \{y \}$ and $B'' = B' \backslash \{y\}$. Note that $A'|B'$ and $A''|B''$ are compatible as $A' \cap B''$ is empty (since $B''$ is a subset of $B' = X \backslash A'$), and therefore $A|B,A'|B'$ and $A''|B''$ are all in the same connected component.

Then, $Int(A''|B'',C|D)=k+1$, as to form $A''$ we removed an element from *A* that was not in *C*, and then added an element that was in *C*. By taking $E|F = A''|B''$, the claim is therefore proven. Hence, *G* is connected.

We will now show that *G* is vertex-transitive.

Let *A*|*B* and *C*|*D* be two vertices of *G*, where $|A|=|C|=m, |B|=|D|=m+1$. Let $\sigma$ be a permutation of *X* so that $\sigma (A)=C$ and $\sigma (B)=D$. Then, the induced action by applying this permutation to every split in *G* is an automorphism that maps *A*|*B* to *C*|*D*; hence, *G* is vertex-transitive.

By Theorem 3.1, as *G* is connected and vertex-transitive, if *G* has an even number of vertices there exists a perfect matching and if *G* has an odd number of vertices, there exists a defect-1 matching.

Finally, the number of vertices of *G* is $$\left( {\begin{array}{c}n\\ m\end{array}}\right) $$, and Corollary 3.1 of Kummer’s Theorem tells us exactly when this value is odd and when it is even. The lemma follows. $\square$

### Example 3.2

Let $X = \{1,2,3,4,5\}$, so $n=5$ and $m=2$. Then, we can partition *Bip*(5, 2) into compatible pairs, for example $\{12|345,34|125\}$,$\{13|245,25|134\}$,$\{14|235,35|124\}$,$\{15|234,24|135\}$ and $\{23|145,45|123\}$. Note that each pair is precisely the set of non-trivial splits corresponding to a unique tree from Fig. 1.

## Minimal Universal Tree Sets

We will shortly prove the main theorem of this paper, Theorem 4.1, pending some useful theorems. The statement of the theorem requires the following definition.

### Definition 4.1

Let *x* be a real number. Then, the ceiling of *x*, denoted by $\left\lceil x \right\rceil$, is the smallest integer *n* so that $n \ge x$. The floor of *x*, denoted by $\lfloor x \rfloor$, is the largest integer *n* so that $n \le x$.

### Theorem 4.1

(Main Theorem) Let *X* be a set of size $n \ge 2$, and let *m* be a positive integer such that $n=2m$ if *n* is even and $n=2m+1$ if *n* is odd. Then, a minimal universal tree set for *X* has size

$$\begin{aligned} U(n) = \left\lceil \frac{1}{2} \left( {\begin{array}{c}n\\ m\end{array}}\right) \right\rceil . \end{aligned}$$

To prove this, we will need a few useful classical theorems from extremal set theory. A *poset* is a set *P* together with a binary relation $\le$ on its elements that is reflexive ($x \le x$ for all $x \in P$), antisymmetric (if $x \le y$ and $y \le x$ then $x=y$ for all pairs $x,y \in P$) and transitive (if $x \le y$ and $y \le z$ then $x \le z$ for all triples $x,y,z \in P$). Define *P*(*X*) to be the poset on the power set of *X*, ordered by set inclusion. In particular, we need a theorem of Sperner and a theorem of Dilworth, which we will use to partition *P*(*X*) into chains, which is necessary for constructing sets of compatible splits.

### Definition 4.2

Let *P* be a poset with a reflexive, antisymmetric and transitive binary relation $\le$ on its elements. Two elements *x* and *y* of *P* are said to be *comparable* if either $x \le y$ or $y \le x$. We call a subset *S* of *P* a *chain* if any two of its elements are comparable, and an *antichain* if no distinct pair of its elements is comparable.

Note that for our example, *P*(*X*), the power set of *X* with the binary relation of set inclusion, a chain is a set *S* of sets in *P*(*X*) so that for any pair of sets *A*, *B* in *S*, either *A* is contained in *B* or *B* is contained in *A*. An antichain in our example is a set *S* of sets in *P*(*X*) so that for any pair of distinct sets *A* and *B*, neither is contained in the other.

### Theorem 4.2

(Sperner (1928)) Let *X* be a set of size *n*. Then, the largest antichain in *P*(*X*) has size $$\left( {\begin{array}{c}n\\ \lfloor n/2 \rfloor \end{array}}\right) $$.

### Theorem 4.3

(Dilworth 1950) Let *P* be a poset and suppose the largest antichain in *P* has size *r*. Then, *P* can be partitioned into *r* chains.

We are now ready for the proof, which we will briefly set up, before dividing into even and odd cases.

### Proof

Fix *n* and let $n=2m$ if *n* is even and $n=2m+1$ if *n* is odd. Now, consider the poset *P*(*X*) where $|X|=n$. Theorem 4.2 states that the largest antichain of *P*(*X*) has size $$\left( {\begin{array}{c}n\\ m\end{array}}\right) $$, and Theorem 4.3 implies that we can therefore partition *P*(*X*) into $$\left( {\begin{array}{c}n\\ m\end{array}}\right) $$ chains. Certainly no subset of size *m* can be contained in a distinct subset of size *m*, so each chain contains at most one subset of size *m*. As there are exactly $$\left( {\begin{array}{c}n\\ m\end{array}}\right) $$ such subsets, each chain must therefore contain exactly one subset of size *m*.

Select any such partition into chains and consider the graph *Chain*(*X*) in which the vertices are the non-empty subsets in *P*(*X*) of size *m* or less, and there is an edge $e=(U,V)$ if and only if

1. $U < V$ in *P*(*X*) and there is no set $W \in P(X)$ where $W \ne U,V$ such that $U< W < V$; and
2. *U* and *V* are elements of the same chain.

Let

$$\begin{aligned} \gamma : V(Chain(X)) \rightarrow {\mathcal {S}}(X) \end{aligned}$$

be the function that maps the subset *A* to the split $A|(X \backslash A)$. Observe that if *n* is even, then $\gamma$ is not injective, and $\gamma (A)=\gamma (B)$ if and only if $A=X \backslash B$. However, if *n* is odd, then $\gamma$ is indeed injective as $X \backslash A$ must have a size larger than *m*. We now split into even and odd cases.

*Case 1* *n* is even Suppose specifically now that *n* is even and define *BipChain*(*X*) to be the graph consisting of vertices $\gamma (V(Chain(X)))$ and an edge $e=(\gamma (U),\gamma (V))$ if and only if $(U,V) \in E(Chain(X))$.

In particular, $\gamma (A)=\gamma (B)$ if and only if $A=X \backslash B$, which occurs precisely when $|A|=m$. Thus, *BipChain*(*X*) has exactly half the number of components that *Chain*(*X*) has, as for each split *A*|*B* the chain in *Chain*(*X*) containing *A* and the chain in *Chain*(*X*) containing *B* are mapped by $\gamma$ to the same component in *BipChain*(*X*).

Hence, the number of components of $\gamma (Chain(X))$ will be

$$\begin{aligned} k = \frac{1}{2} \left( {\begin{array}{c}n\\ m\end{array}}\right) . \end{aligned}$$

We construct a universal tree set as follows. Denote the components of $\gamma (Chain(X))$ by $C_1,\ldots ,C_k$.

We claim that the set of phylogenetic trees corresponding to the sets of splits $V(C_1),\ldots ,V(C_k)$ via the Splits Equivalence theorem, is a universal tree set, in particular that all $V(C_i)$ are sets of compatible splits.

First, let the unique split of size *m* in $V(C_i)$ be *A*|*B*. Suppose we have two distinct splits, *C*|*D* and $C'|D'$. In order to show compatibility, it suffices (but is not necessary) to show that one of *C* or *D* is contained in one of $C'$ or $D'$, or the reverse, as the inclusion requirement quickly holds.

If $C'|D' = A|B$, then certainly *C* or *D* is a subset of *A* or *B* by the chain construction. We therefore assume neither split is *A*|*B*, and without loss of generality that $|C| < |D|$ and $|C'| < |D'|$. Then, either *C* and $C'$ are subsets of the same set (*A* or *B*), or one is a subset of *A* and the other of *B*. If *C* and $C'$ are subsets of the same set, then *C* and $C'$ are from the same component of *Chain*(*X*) and therefore $C \subseteq C'$ or the reverse. However, if they are subsets of different sets, then $C \subset X \backslash C' = D'$. Therefore, in all cases $V(C_i)$ is a set of compatible splits, and as every split is present in some $V(C_k)$ the tree set corresponding to $V(C_1),\ldots ,V(C_k)$ via the Splits Equivalence theorem is a universal tree set.

It finally remains to confirm that

$$\begin{aligned} k = \left\lceil \frac{1}{2} \left( {\begin{array}{c}n\\ m\end{array}}\right) \right\rceil . \end{aligned}$$

is the minimum possible value. However, as each phylogenetic tree in any universal tree set can contain at most one split of size *m* by Lemma 3.1 (of which there are $$\left( {\begin{array}{c}n\\ m\end{array}}\right) /2$$, which is equal to the desired formula when *n* is even), the set is minimal. The theorem follows in the even case.

The proof for odd *n* proceeds similarly, with some small modifications.

*Case 2* *n* is odd

Suppose specifically now that *n* is odd. We perform one additional modification, relative to the even case. By Lemma 3.3, there exists a matching *M* between the splits of the form *A*|*B* where $|A|=m, |B|=m+1$ with at most one unpaired split. For each of the

$$\begin{aligned} \left\lfloor \frac{1}{2} \left( {\begin{array}{c}n\\ m\end{array}}\right) \right\rfloor \end{aligned}$$

such matchings (*A*|*B*, *C*|*D*) in *M*, add this edge to $\gamma (Chain(X))$, and call the resulting graph *BipChain*(*X*).

As $\gamma (Chain(X))$ contained $$\left( {\begin{array}{c}n\\ m\end{array}}\right) $$ connected components and each additional edge reduced the number of components by one, the number of connected components of *BipChain*(*X*) will therefore be

$$\begin{aligned} k = \left( {\begin{array}{c}n\\ m\end{array}}\right) - \left\lfloor \frac{1}{2} \left( {\begin{array}{c}n\\ m\end{array}}\right) \right\rfloor = \left\lceil \frac{1}{2} \left( {\begin{array}{c}n\\ m\end{array}}\right) \right\rceil . \end{aligned}$$

We construct a universal tree set as follows. Denote the components of $\gamma (Chain(X))$ by $C_1,\ldots ,C_k$.

We claim that the set of phylogenetic trees corresponding to the sets of splits $V(C_1),\ldots ,V(C_k)$ (via the Splits Equivalence theorem) is a universal tree set, and in particular that all $V(C_i)$ are sets of compatible splits.

Let the two (distinct) splits of size *m* in $V(C_i)$ be *A*|*B* and $A'|B'$, and let *C*|*D* and $C'|D'$ be any two distinct splits in $V(C_i)$. If *C*|*D* and $C'|D'$ are in the same component in *Chain*(*X*), the options proceed analogously to the even case, and *C*|*D* is compatible with $C'|D'$.

Therefore, instead suppose that *C*|*D* was in the same component of *Chain*(*X*) as *A*|*B* and $C'|D'$ was in the same component of *Chain*(*X*) as $A'|B'$. In particular, without loss of generality suppose that $C \subseteq A$ and $C' \subseteq A'$.

As *A*|*B* and $A'|B'$ are compatible, one of $A \cap A', A \cap B', B \cap A'$ or $B \cap B'$ are empty, but as *B* and $B'$ have size $m+1$ the only possibility is that $A \cap A'$ is empty. Then, as $C \subseteq A$ and $C' \subseteq A'$ it follows that $C \cap C'$ is empty, so *C*|*D* and $C'|D'$ are compatible.

It finally remains to confirm that

$$\begin{aligned} k = \left\lceil \frac{1}{2} \left( {\begin{array}{c}n\\ m\end{array}}\right) \right\rceil . \end{aligned}$$

is the minimum possible value. By Lemma 3.2 in each phylogenetic tree in a universal tree set, there can be at most two splits of size *m*, of which there are $$\left( {\begin{array}{c}n\\ m\end{array}}\right) $$, implying that

$$\begin{aligned} k = \left\lceil \frac{1}{2} \left( {\begin{array}{c}n\\ m\end{array}}\right) \right\rceil \end{aligned}$$

is a lower bound (noting that the binomial coefficient can be odd, hence the ceiling function). $\square$

### Remark 4.1

One can observe that the universal tree sets constructed in the proof of Theorem 4.1 are highly non-unique, as they depend entirely on the partition into chains at the beginning of the proof. However, all trees in the constructed universal tree set are either caterpillar trees or have a binary refinement that is a caterpillar tree, as a consequence of the construction.

We note here that for *U*(*n*), the associated integer sequence ($3,5,10,18,35,63\ldots$) appears in the OEIS as sequence A002661 for $n \ge 4$ (OEIS Foundation Inc. 2020), in which it appears as the sequence of least integers having Radon random number *n*.

## Applications and Discussion

A split network is a combinatorial generalisation of a tree in which sets of edges are associated with splits instead of just a single edge (Huson and Bryant 2006). Due to this, each split network is associated with a split system (which is not necessarily pairwise compatible) and so give us a rich source of split systems for which we can find minimal tree sets. Indeed, Huson and Bryant (2006) state that the interpretation of split networks relies on a single principle: “a split network contains exactly the same information as a list of splits [with a weight for each split]” . In this sense, the interpretation of our main result applied equally to all types of split networks, for example the median networks of Bandelt et al. (1999) and the consensus networks of Holland and Moulton (2003)—we have shown an upper bound for the possible complexity of a split network. In fact, Holland and Moulton construct their consensus networks with explicit reference to *k*-compatibility rather than minimal tree sets, and it would be an interesting research direction to consider how their results change in this new context.

**Fig. 2:**
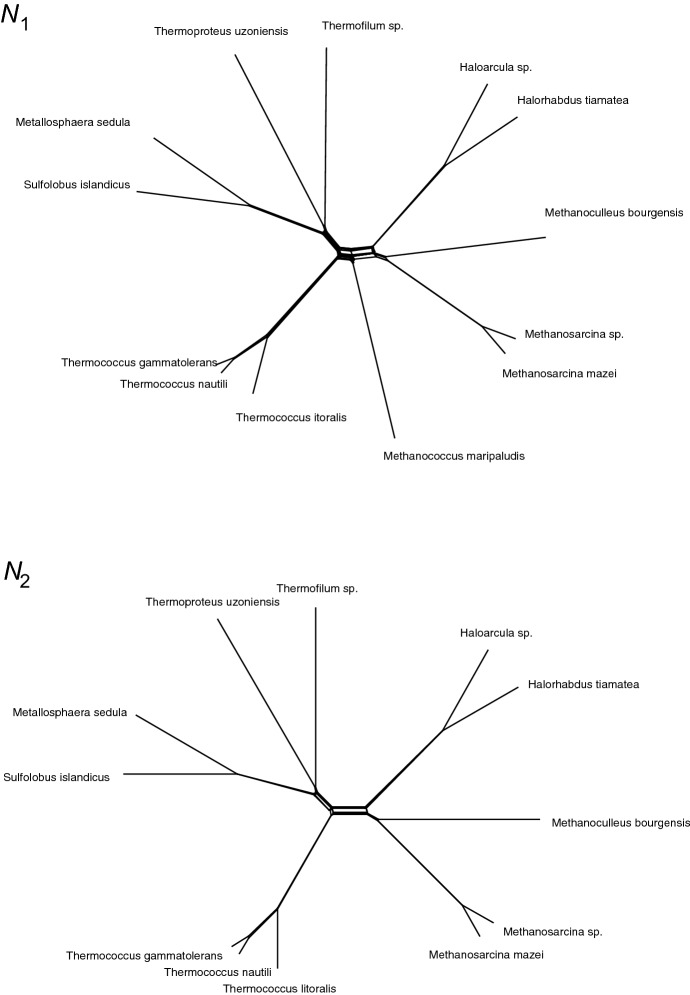
Networks $N_1$ and $N_2$ derived from archaeal genomes

We now consider two SplitsTree-generated (Huson and Bryant 2006) split networks derived from archaeal genomes and depicted in Fig. 2. The first network $N_1$ contains 13 taxa, and the second network, $N_2$, is the network obtained after removal of the single taxon *Methanococcus maripaludis*, leaving 12 taxa. We note here that this deviates from the main perspective of this manuscript, as we are now considering two split systems on explicitly different sets of taxa. However, our intention is to use the main result of the previous section to normalise the minimal tree set sizes corresponding to these networks, and the difference in taxa sets is precisely what makes this normalisation necessary.

To generate these networks, 39 universal archaeal protein families gathered by Nelson-Sathi et al. (2015) were used for a BLAST search against archaeal genomes obtained from the RefSeq 2016 database (O’Leary et al. 2016) with an identity threshold of 20% and an e-value cut-off of $10^{-5}$ (The e-value indicates the number of expected hits with a similar score that could be found just by chance. It is a measure to identify significant hits in a dataset). For each best hit, alignments were generated using MAFFT v7.299b (linsi) (Katoh et al. 2002) and concatenated using an in-house Python script. These concatenated alignments were used to construct a Neighbor-Net using SplitsTree4. We note here that all splits represented by the data are also present in the networks shown in Fig. 2. The source files are available as supplementary data, including the in-house script and the distance matrix used to construct the Neighbor-Net.

Using a short Python program that we have made available online (Hendriksen 2020), we analysed the splits corresponding to each network and found a set of 4 phylogenetic trees that display all splits in $N_1$, and a set of 3 phylogenetic trees that display all splits in $N_2$. We note that the splits were analysed from the source file, so necessarily included all splits indicated by the data. These were then shown to be minimal by hand—both networks display 3 incompatible splits of size 6, and $N_1$ additionally displays a partition *A*|*B* of size 5 that is incompatible with each of the first 3. Therefore, if we denote the minimal tree set size of the split system associated with a network *N* by $\kappa (N)$, we know $\kappa (N_1)=4$ and $\kappa (N_2)=3$. It was observed that there are several possible minimal tree sets that can be computed for the split systems corresponding to $N_1$ and $N_2$, but this of course does not affect the values of $\kappa (N_1)$ and $\kappa (N_2)$.

If we denote the number of leaves of a network *N* by |*N*|, then we can define the *normalised tree set size* $\mathrm{Norm}(N)$ to be

$$\begin{aligned} \mathrm{Norm}(N)=\frac{\kappa (N)}{U(|N|)}. \end{aligned}$$

Now, as $U(13)=858$ and $U(12)=462$, we can normalise these minimal tree set sizes, and as

$$\begin{aligned} \mathrm{Norm}(N_1) = \frac{4}{858} < \mathrm{Norm}(N_2)= \frac{3}{462}, \end{aligned}$$

from the perspective relative to universal incompatibility, $N_2$ is ‘more incompatible’ than $N_1$.

Although the underlying dataset consists of Archaeal proteins, which are known to have lateral gene transfer events (Nelson-Sathi et al. 2015), the specific proteins which are used here are mainly ribosomal proteins. Ribosomal subunits are involved in the cellular process of translation. It is known that they are very conserved proteins across all life forms (Ban et al. 2014). We deliberately made the choice to examine Archaea given these lateral gene transfer events, so that we could expect some discordance, but selected ribosomal proteins to limit the extent thereof for ease of analysis.

The organism which is removed from $N_1$ to obtain $N_2$, *Methanococcus maripaludi*, is a fully sequenced model organism among hydrogenotrophic methanogens (Goyal et al. 2016) and is the only member of the genus *Methanococcus* in our dataset.

As the only organism of the genus *Methanococcus*, we would expect the *Methanococcus* to be evolutionarily distinct from the remaining organisms. We would therefore predict that it would contribute proportionately less to the incompatibility of the data with respect to the remaining twelve organisms of the dataset, so the result that $N_2$ is relatively more incompatible than $N_1$ is as anticipated.

Mathematically speaking, there are several natural extensions to the problem of minimal universal tree sets for future research. For instance, one avenue could be to investigate how the value changes if we instead ask for a minimal universal set of networks with at most *k* reticulations. The probabilistic analogue of the question would also be interesting—how likely is it, given a set of *k* trees, to have a universal tree set (in particular for the minimal case, $k= U(n)$)?

There are several natural combinatorial questions that can also be asked. For instance, we could define a generalisation of universal incompatibility *U*(*n*, *k*), in which rather than requiring every split in ${\mathcal {S}}(X)$ to be represented in our tree set, we require only that all splits of size *k* or less are displayed by a tree in the set (with the present paper of course corresponding to the case $k = \lfloor \frac{n}{2} \rfloor$). One may also consider other important split sets in place of ${\mathcal {S}}(X)$, such as the set of all splits *A*|*B* in which some given subsets $A',B' \subset X$ must be placed in different partitions, that is, either $A' \subset A$ and $B' \subset B$ or $A' \subset B$ and $B' \subset A$.
